# Immune Cells in the Placental Villi Contribute to Intra-amniotic Inflammation

**DOI:** 10.3389/fimmu.2020.00866

**Published:** 2020-05-22

**Authors:** Jessica M. Toothaker, Pietro Presicce, Monica Cappelletti, Stephanie F. Stras, Collin C. McCourt, Claire A. Chougnet, Suhas G. Kallapur, Liza Konnikova

**Affiliations:** ^1^Department of Immunology, University of Pittsburgh, Pittsburgh, PA, United States; ^2^Divisions of Neonatology and Developmental Biology, David Geffen School of Medicine at the University of California, Los Angeles, Los Angeles, CA, United States; ^3^Division of Newborn Medicine, Department of Pediatrics, UPMC Children's Hospital of Pittsburgh, Pittsburgh, PA, United States; ^4^Division of Immunobiology, Cincinnati Children's Hospital Research Foundation, University of Cincinnati College of Medicine, Cincinnati, OH, United States; ^5^Department of Developmental Biology, University of Pittsburgh, Pittsburgh, PA, United States; ^6^Department of Pediatrics, Yale University, New Haven, CT, United States

**Keywords:** choriodecidua, CyTOF, immune, intra-amniotic inflammation, placental villi

## Abstract

Intra-amniotic (IA) inflammation is associated with significant morbidities for both the mother and the fetus. Prior studies have illustrated many of the effects of IA inflammation on the uterine lining (decidua) and membranous layers of the placenta at the fetal–maternal interface. However, much less is known about the immunological response occurring within the villous placenta. Using a rhesus macaque model of lipopolysaccharide (LPS)-induced IA inflammation, we showed that pregnancy-matched choriodecidua and villi have distinct immunological profiles in rhesus pregnancies. In the choriodecidua, we show that the abundance of neutrophils, multiple populations of antigen-presenting cells, and two populations of natural killer (NK) cells changes with prenatal IA LPS exposure. In contrast, in immune cells within the villous placenta we observed alterations in the abundance of B cells, monocytes, and CD8 T cells. Prior work has illustrated that IA inflammation leads to an increase in tumor necrosis factor alpha (TNFα) at the fetal–maternal interface. In this study, pretreatment with a TNFα blockade partially reversed inflammation in the placental villi. Furthermore, we report that immune cells in the villous placenta sensed LPS during our experimental window, and subsequently activated T cells to produce proinflammatory cytokines. Moreover, this study is the first report of memory T cells in third-trimester non-human primate placental villi and provides evidence that manipulation of immune cells in the villi at the fetal–maternal interface should be considered as a potential therapeutic target for IA inflammation.

## Introduction

Intra-amniotic (IA) inflammation is believed to be driven by both pathogenic and sterile inflammatory processes (1). Downstream morbidities of IA inflammation include placental dysfunction, intrauterine growth restriction, preeclampsia, and spontaneous preterm labor. Each of these pathologies is also associated with preterm birth, currently the number one cause of mortality in children under 5 years of age (2). Furthermore, premature infants exposed to IA inflammation are at even higher risk of developing further comorbidities including periventricular leukomalacia and retinopathy of prematurity than age-matched premature infants without IA inflammation (3, 4). Although IA inflammation is restricted to the fetal–maternal interface and the amniotic fluid, pathogen-associated IA inflammation leads to inflammatory responses in multiple other fetal organs including the brain, lungs, skin, and gut in humans and animals (5, 6). Much of the work investigating infection-driven inflammation at the fetal–maternal interface has focused on the maternally derived decidua and/or the fetus-derived fetal membranes (7–11). Yet, the fetus-derived placental villi, which is in contact with the fetal membranes and invades into the maternal decidua, comprises the bulk of the fetal–maternal interface by mass. The villi participate in the exchange of nutrients and gases between the fetus and mother, and potentially contribute to inflammation (12). Therefore, the role played by villi-derived immune cells during IA inflammation should be investigated further. It has been shown that leukocytes isolated from villous blood are functionally different than those in the general circulation (13). For example, placental immune cells from non-laboring patients transcribe less interleukin (IL)-1β than immune cells in the circulation at baseline (13). Moreover, immune cells that are distinct from those in fetal and maternal blood have been found within the villous tissue (14). However, the complete immune landscape within the placental villi and its potential contribution to IA inflammation remains poorly understood.

It is now appreciated that human fetuses can generate phenotypical memory T cells as early as the second trimester (15–17). Furthermore, these tissue-resident memory T cells, and other fetal immune cells, secrete pro-inflammatory cytokines even in healthy pregnancies (15–20). As further evidence for *in utero* memory T-cell generation, it is reported that noninherited maternal antigens generate a T-cell response in a fetus, a pathway particularly important in the generation of T regulatory cells (Tregs) (21). Additionally, a proportion of T cells extracted from cord blood of preterm infants secrete tumor necrosis factor-alpha (TNFα) and interferon-gamma (IFNγ) when exposed to case-matched, but not unmatched, maternal blood antigens, suggestive of a memory phenotype (19). Moreover, a recent single-cell sequencing analysis revealed the presence of both maternal and fetal activated T cells within the villi of term and preterm placentas (22). This work leads us to hypothesize that fetal derived placental villi contain functional immune cells that contribute to IA inflammation in a tissue-specific manner.

It is well-documented that there are anatomic and physiologic differences between the villi of murine and primate placentas preventing mice from being good surrogate models (23, 24). Although mice and humans both have hemichorial placentas, with maternal blood in direct contact with fetal cells, human villi are tree-like, float in maternal blood, and anchor deeply into the maternal decidua. In contrast, the mouse placenta is organized into a labyrinth architecture; maternal blood flows in an organized fashion into the labyrinth, and there is a junctional zone separating fetal villi from maternal decidua (24). Therefore, studies that use models more like humans, such as non-human primates, to investigate the role and function of placental immune cells are needed (24, 25). As such, we have used a non-human primate model of LPS-induced IA inflammation (26). This model induces high levels of TNFα within the choriodecidua (8); for this reason we elected to study the effects of TNFα specifically and examined whether the effect of pretreatment with a TNFα blockade impacts IA inflammation.

Using mass cytometry (CyTOF) to profile the immune cells derived from pregnancy-matched choriodecidua and placental villi, we showed that the immune cells within the villi are distinct from those in the neighboring choriodecidual layer. This finding supported our hypothesis that immune responses at the fetal–maternal interface during IA inflammation are tissue specific. In our model, LPS induced both STAT1 and IRAK4 phosphorylation in villi antigen presenting cells (APCs). Furthermore, IA LPS was able to alter the cytokine production of and induce activation (e.g., HLA-DR^+^) of T cells in a TNFα-dependent manner. Finally, IA LPS induced a reduction in Tregs in the villi, a phenomenon that could be responsible for the overactivation of T cells seen in our model. This work illustrates the previously underappreciated role of immune cells in the primate placental villi as players in the inflammatory environment of infectious preterm deliveries and should be considered in therapies for prevention of IA inflammation.

## Materials and Methods

### Animals

Adult multiparous female rhesus macaques (*Macaca mulatta*) (*n* = 14) were time mated at the California Primate Center, UC Davis. At ~130 days (~80%) of gestation the pregnant dam received either 1 mL of saline solution (*n* = 4) or 1 mg of LPS (Sigma-Aldrich, St. Louis, MO, *n* = 5) in 1 mL of saline solution by ultrasound-guided IA installation. Two of the four monkeys received intramuscular saline instead of IA; however, no LPS was administered intramuscularly. The TNF blocker adalimumab (Humira, AbbVie Inc. North Chicago, IL) alone was administered to the blockade group by IA (40 mg) + maternal subcutaneous (40 mg) at 1 and 3 h before LPS (*n* = 5) to inhibit TNF signaling in the amniotic and systemic compartments (Figure S1). The concentration of 1 mg of LPS was determined based on prior experiments using a sheep model of LPS-induced IA inflammation (27). One milligram was then calculated as an allometric derivation, proportional to the average rhesus weight compared to the amount used for the average weight of sheep. Fetuses were delivered via Cesarean section 16 h after LPS injection. All animals were monitored by veterinarians through anesthetization and surgical procedures. Additionally, monkeys were monitored extensively for 7 days post C-section for food intake, fecal composition, hydration, activity, and overall discomfort including monitoring of incision sites. A 16-h duration was determined to be optimal in prior studies using the same method of LPS-driven inflammation in pregnant rhesus macques (8). The maternal macaques and their fetuses were similar in clinical variables (Table S1). After delivery, fetuses were euthanized with pentobarbital, and fetal tissues were collected. There were no spontaneous deaths or preterm labor in each animal group. As the experimental window was started at 80% gestation, the delivered fetuses were still premature neonates, though there was no preterm labor. All animal care and experiments were approved by the National Primate Center and were in accordance with University of California Davis IACUC protocol #20330.

### Placental Dissection

Extraplacental membranes were dissected away from the placenta as previously described (7, 8). After decidua parietalis cells with the attached chorion were dissected and removed, the amnion, decidua basalis, and rest of the chorionic tissue were peeled away from each other with forceps. After full separation of membranes and decidua, portions of villous tissue were isolated and shipped overnight on ice to the University of Pittsburgh. Chorio-decidua cells representing decidua parietalis with attached chorion were washed and digested with Dispase II (Life Technologies, Grand Island, NY) plus collagenase A (Roche, Indianapolis, IN) followed by DNase I (Roche) treatment, as previously described (7, 8). Villi samples were digested identically to choriodecidua with the substitution of Accutase (Millipore) in place of Dispase II at the same concentration and timing.

### CyTOF Staining

*Phosphostaining* Single-cell suspensions were divided evenly among condition groups. Phosphostained samples were incubated for 20 min under 37°C and 5% CO_2_ conditions with no *ex vivo* stimulation. Post 20-min incubation, cells were stabilized through fixation using 0.2% paraformaldehyde and then washed with cell staining buffer (CSB), which consists of DPBS with 0.5% bovine serum albumin (Sigma) and 0.02% sodium azide. After washing, cells were incubated with human TruStain FcX (Biolegend) The metal-coupled surface staining antibodies were then applied to each sample (0.5 μL of each antibody per tube). Cells were then washed twice with CSB and fixed with 1.6% paraformaldehyde. Ice-cold methanol was used to permeabilize the cells that were washed with CSB. Metal-coupled intracellular staining antibodies were added, and the cells were washed twice with CSB.

*Cytokine Staining* Cells were incubated with equivalent concentrations GolgiStop/GolgiPlug as in Photostaining above but incubated for 4 h at 37°C and 5% CO_2_ with no *ex vivo* stimulation. Cells were washed in CSB after surface staining (as above), incubated in FOXP3 fixation and permeabilization solution (Invitrogen), washed with 1 × FOXP3 wash buffer (Invitrogen), and stained with an intracellular antibody cocktail.

*All Staining* After intracellular staining, cells were resuspended in 1.6% paraformaldehyde and washed with CSB. Samples were stored in Freezing Media (FBS + 10% DMSO) until the day of CyTOF run. Freezing media was centrifuged and cells were washed with CSB. Cells were labeled with intercalator-Ir (Fluidigm). Intercalator solution was centrifuged and cells were washed twice with CSB. Villi: Samples were then shipped overnight with refrigeration to the Longwood Medical Area CyTOF Core of the Dana-Farber Cancer Institute. Choriodecidual samples were run on site at the University of California, Los Angeles Flow Cytometry Facility. Before being run on the Helios (Fluidigm) CyTOF machine, villi samples were resuspended in MilliQ water with a 1:10 dilution of EQ beads for normalization.

### Data Analysis

Bead normalized FCS files were uploaded to Premium Cytobank®, where they were pregated on DNA^+^, live (Rh103^−^), bead^−^ prior to subsequent analysis. Percent positive and mean metal intensity (MMI) parameters were exported from 2D gated populations within Premium Cytobank software. Samples with insufficient intracellular staining were omitted from individual analyses (Table S2). Data were then uploaded to either R or GraphPad Prism software for analysis and graph generation. For automatic clustering, parent populations as indicated in figures were 2D gated and exported from Cytobank and then uploaded to the cytofkit R package (doi: 10.18129/B9.bioc.cytofkit) (28–30). Samples with insufficient cell number were omitted from analysis (Table S2). Data were transformed with cytofAsinh and merged with ceil. *t*-distributed stochastic neighbor embedding (tSNE) was used for dimensionality reduction. Phenograph was selected from clustering for all data using the preset *k* = 30. All data were visualized using the https://cytofkit.shinyapps.io/shiny/platform. All abundance data displayed in the article were extracted from the Phenograph clustering output. Cluster identification was achieved using generated heatmaps for antibody intensity. Any antibodies that showed insufficient staining across all samples were omitted from heatmaps.

*Statistical Analysis and Plot Generation* R version 3.5.1 was used to perform statistical analysis between treatments using the Kruskal–Wallis analysis and *post hoc* analysis with Dunn's multiple comparison test because of the non-parametric nature of primate data using PMCMR and data.table packages; *P* < 0.05 was considered significant. Plots were generated in Prism GraphPad 7. Each data point represents one monkey unless otherwise indicated.

### Data and Code Availability

The data analyzed in this study are stored in accordance with Institutional Animal Care and Use Committee (IACUC) guidelines. Requests can be directed to Liza Konnikova ( Liza.konnikova@chp.edu).

## Results

### The Villous Placenta Has a Distinct and Diverse Immune Landscape

**Figure 1:**
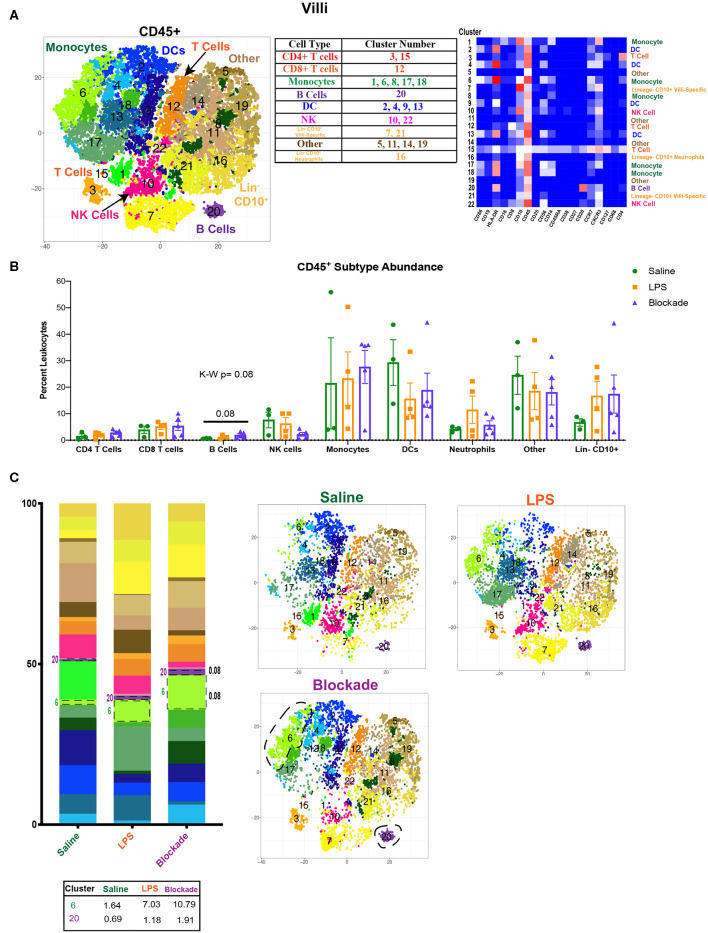
Immune landscape of healthy and inflamed villi. **(A)** Automated clustering of CD45^+^ cells isolated from the villi. tsne is a merged image of all monkeys [saline (*n* = 3), LPS (*n* = 4), blockade (*n* = 5)] analyzed. Clusters were identified as one of nine major immune subtypes by antibody intensity represented by heatmap. **(B)** Abundance of each major subtype listed in the table in **(A)**. Kruskal–Wallis values are listed at the top of each graph. *Post hoc P*-values are listed as lines connecting respective comparisons. **(C)** tsne plots and stacked bar graphs of cluster abundance separated by treatment group. Outlined populations are those that are altered between treatment groups. Mean percentage of these clusters are listed in the table that follows. Kruskal–Wallis values are listed next to each cluster with statistical trends.

To determine the effects of inflammation on the villous placenta, we investigated the immunophenotype and activation of immune cells within the tissue and the impact of prenatal exposures. Villous cells were isolated using previously established protocols (5, 6) from pregnant rhesus dams exposed to 16 h of either IA saline or intramuscular (IM) saline (*n* = 4), LPS (*n* = 5), or pretreatment with TNFα inhibitors and LPS (*n* = 5), referred to as the blockade group throughout the article (Figure S1A, Table S1). To determine the immune landscape of villi, we performed CyTOF using a panel of 22 surface antibodies (Table S3). To determine the global immune profile, we first clustered on all leukocytes (CD45^+^) and identified populations based on surface marker expression (Figure 1A). We observed a complex immune landscape and identified cells as follows: including monocytes/macrophages (CD14^+^, which will be referred to as monocytes in this article), natural killer (NK) cells (CD14^−^CD19^−^CD4^−^ CD8^−^ HLA-DR^−^CD56^+^), dendritic cells (DCs) (CD14^−^CD19^−^CD4^−^CD8^−^ HLA-DR^+^), B cells (CD14^−^CD19/CD20^+^), T cells (CD14^−^CD19–^−^CD4/CD8^+^), innate lymphoid cells (ILCs) (CD14^−^CD19^−^CD4^−^ CD8^−^ HLA-DR^−^CD56^−^CD127^+^), and presumptive neutrophils or presumptive hematopoietic stem cells (CD14^−^CD19^−^CD4^−^CD8^−^ HLA-DR^−^CD56^−^CD10^+^) (Figure 1A).

When examining the CD45^+^ population, we found a complex and highly diverse immune landscape in the villous placenta that has not been previously appreciated (Figure 1A). Notably, the villous placenta was composed not only of monocytes (Hofbauer cells), whose presence and role during pregnancy have been previously described (31–33), but also contained DCs (10%−40%), indicating multiple APC populations within the tissue. Moreover, we observed a group of clusters that were lineage^−^ and CD10^+^. These cells likely represent hematopoietic stem cells (34, 35). Unfortunately, we were unable to further phenotype these cells with our current panel. We observed no changes in population abundance when all clusters of each major immune subtype were grouped together (Figure 1B). However, we did see a trend toward an increase in the blockade group (*P* = 0.08) in cluster 20, the only B-cell population found in the villous placenta (Figures 1B,C). This finding is unique, as B-cell abundance is reported to be very limited in the decidua (36). We also saw a trend (*P* = 0.08) toward a higher abundance of cluster 6 (CD86^+^CD10^+^CCR7^+^) monocytes in IA LPS exposed monkeys that was TNFα independent (Figure 1C).

**Figure 2:**
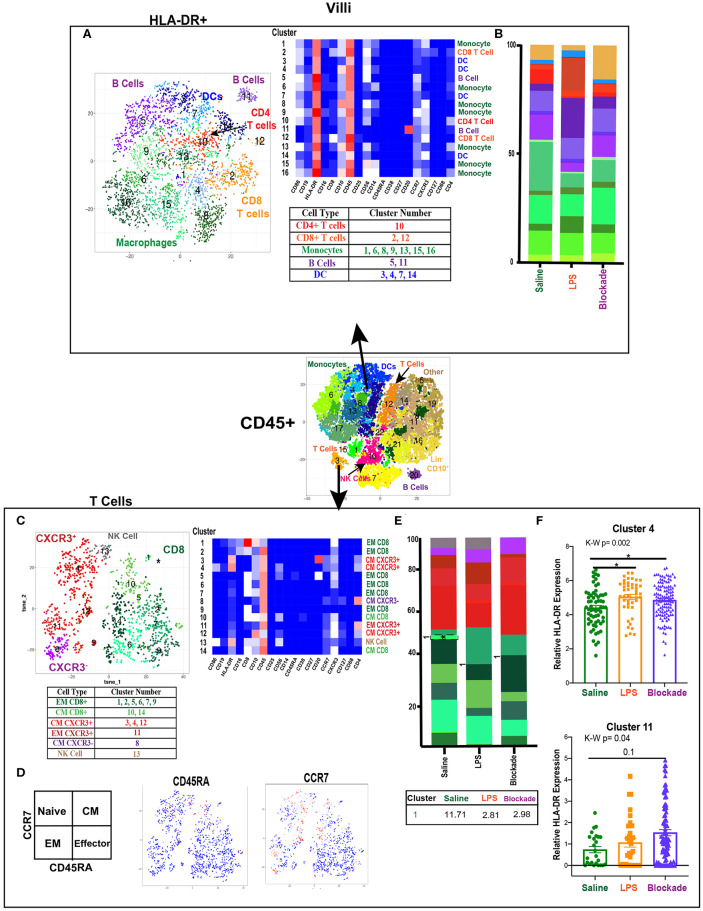
High diversity in villi APC and T cell populations. **(A)** Automated clustering of HLA-DR^+^ cells in the villi, merged tsne of saline (*n* = 3), LPS (*n* = 4), and blockade (*n* = 5). Clusters were identified by relative intensity of each antibody in the heatmap and classified as listed in the subsequent table. **(B)** Stacked bar graphs represent abundance of each cluster within a group. No significant changes were detected in HLA-DR^+^ cells. **(C)** Automated clustering of T cells within the villi saline (*n* = 3), LPS (*n* = 4), and blockade (*n* = 5). CD4 T cells were categorized and labeled on tsne by CXCR3 expression. **(D)** Memory phenotypes determined by CCR7 and CD45RA expression illustrated on tsne. Red = high expression, blue = low expression. **(E)** Stacked bar graphs represent abundance of each cluster within a group. Significantly changed clusters are outlined in black and mean percentage listed in the table below. **(F)** HLA-DR expression in two clusters of T cells, each dot represents a single cell within a treatment group. Kruskal–Wallis values are listed at the top of each graph. *Post hoc P*-values are listed as lines connecting respective comparisons. *P < 0.05.

To determine if there were immune differences at a more granular level, we examined major immune populations separately. To assess APCs and activated T cells in the villi, we clustered on HLA-DR^+^ CD45^+^ cells (Figure 2A). We detected myeloid derived cells (MDCs), B cells, and T cells (Figure 2A). The presence of both APCs and HLA-DR^+^ T cells in the saline group indicated the potential for antigen presentation within the villi in healthy pregnancies. However, these HLA-DR^+^ immune populations remained stable when exposed to IA LPS (Figure 2B).

### Villi T Cells Show Predominantly a Memory Phenotype

When clustering on T cells specifically, we detected both CD4 and CD8 populations, consistent with prior reports (13, 14) (Figure 2C). However, we were able to greatly expand upon these findings and deeply phenotype the T cells within the villous tissue. We showed that in saline-treated placentas, the T-cell compartment was made up of 56% CD8, 38% CD4, and 6% NK/NKT cells. Additionally, within the CD4 populations we report most clusters were CXCR3^+^, suggesting a T_H1_ phenotype (Figure 2C). Using expression of CCR7 and CD45RA, we found that in all treatment groups there were no clusters segregated as naïve (CCR7^+^CD45RA^+^) T cells of either CD4 or CD8 phenotype (Figure 2D). Furthermore, we report that while the majority of CD4 cells (58%) were of central memory (CM) phenotype (CCR7^+^CD45RA^−^), the majority (64%) of CD8 cells were of effector memory (EM) phenotype (CCR7–CD45RA^−^) (Figure 2C). These findings suggest that CD4 T cells were predominately being educated in the vasculature, while CD8 T cells were educated within the tissues. IA LPS had no effect on the relative proportions of T cell populations except for a significant decrease in cluster 1 (CD8^+^CXCR3^+^CD10^+^CD16^+^) EM CD8 T cells in a TNFα-dependent manner (Figure 2E). HLA-DR expression within two CD4 T cell clusters was upregulated with LPS in a TNFα-independent manner [significantly in cluster 4 (CM CXCR3^+^) and trending toward increase in cluster 11 (EM CXCR3^+^)], suggesting that the IA LPS leads to activation of villi CD4 T cells (Figure 2F).

### The Villous Immune Landscape Is Distinct From the Choriodecidua

**Figure 3:**
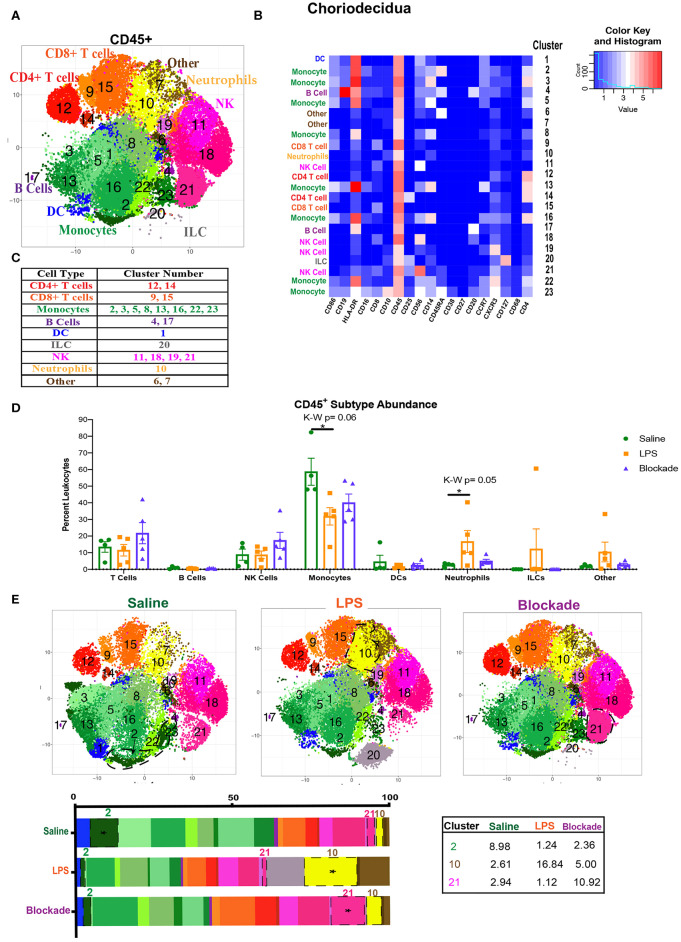
Global immune landscape of healthy and inflamed choriodecidua. **(A)** Automated clustering of CD45^+^ cells isolated from the choriodecidua. tsne is a merged image of all monkeys analyzed saline (*n* = 4), LPS (*n* = 5), and blockade (*n* = 5). **(B)** Clusters were identified as one of nine major immune subtypes based on intensity of antibody staining represented by the heatmap. **(C)** Determination of each cluster as a major immune subset. Kruskal–Wallis values are listed at the top of each graph. *Post hoc P*-values are listed as lines connecting respective comparisons. **(D)** Abundance of each major immune subtype classified in **(C)** as a percent of CD45^+^ cells. **(E)** tsnes and stacked bar graphs of cluster abundance separated by treatment group. Outlined populations are those that are altered between treatment groups. Mean percentages of these clusters are listed in the adjacent table. Tables *P < 0.05.

We next examined the immune compartment in the choriodecidua to determine if the immune landscape reported in the foregoing was villi specific. When looking at the choriodecidual CD45^+^, we observed many immune populations that were either absent from the villi, such as ILCs, or in greatly different abundances. For example, while the saline choriodecidua contained 25–30% T cells (Figure 3D), the villi, in contrast, were only 10–15% T cells (Figure 1B). In the choriodecidua, we observed one population of neutrophils with the phenotype of major lineage marker^−^ CD10^+^, cluster 10 (Figures 3A,B). In the villi, cluster 16 (Figure 1A) is probably also neutrophils, as we saw a similar trend of an increased abundance in LPS-treated animals that is corrected with the blockade (Figure 1C). However, two villi clusters, 7 and 21, with similar surface marker expression (major lineage marker^−^ CD10^+^) were not detected in the choriodecidua (Figures 3A,B). These findings suggest that the diverse immune landscape we found in the villi is tissue specific and unique from the neighboring choriodecidua.

### Choriodecidual Immune Cells Are Sensitive to IA LPS in a TNFα-Dependent Manner

To compare major populations in the choriodecidua, i.e., monocytes, DCs, T cells etc., we combined clusters of the same subtype together (Figure 3C) and saw no significant differences between treatment groups except for monocytes and neutrophils (Figure 3D). However, when looking at individual clusters of immune cells, we observed that there was a significant decrease in abundance of cluster 2 monocytes (CD14^+^HLA-DR^+^CD16^+^CD4^+^CD45RA^+^) in LPS, which was partially restored with the blockade (Figure 3E). Moreover, there was a significant influx of cluster 21 NK cells (CD56^+^CD25^+^CD8^+^), observed specifically with the blockade treatment (Figure 3E). Furthermore, cluster 10, presumptive neutrophils, which also represents the entire neutrophil population (Figures 3C,D), increased significantly with LPS treatment, and was restored back down to normal levels with the blockade (Figure 3E), consistent with previously published work (7, 8). This result suggests that IA LPS results in alterations of the frequency of specific immune populations (i.e., individual clusters). Moreover, TNFα blockade before IA LPS restores some, such as neutrophils, but not all the LPS-induced immune dysregulation.

### Diverse T Cell Populations Are Present in the Choriodecidua

**Figure 4:**
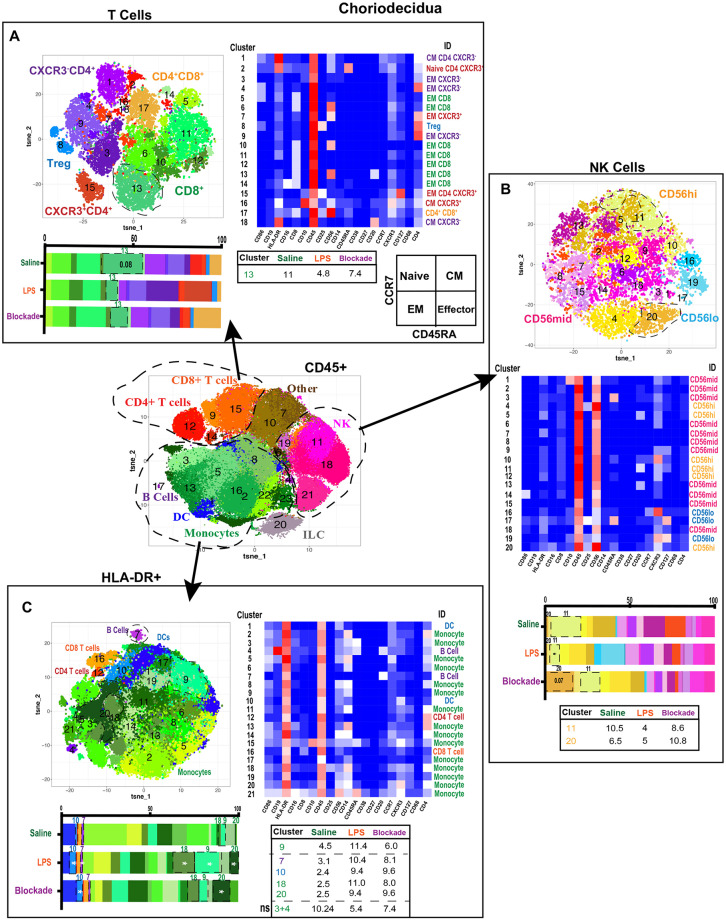
Greater diversity of choriodecidual immune cells was observed with more specific clustering. **(A)** Automated clustering of T cells in the choriodecidua, merged tsne of saline (*n* = 4), LPS (*n* = 5), and blockade (*n* = 5). Clusters were identified by relative intensity of each antibody in the heatmap. Clusters that changed with treatment are outlined in black and mean percentage listed in the adjacent tables. Memory state was determined using CCR7 and CD45RA. **(B)** Automated clustering of NK cells in the choriodecidua, merged tsne of saline (*n* = 4), LPS (*n* = 5), and blockade (*n* = 5). Clusters were identified by relative intensity of each antibody in the heatmap. Clusters that changed with treatment are outlined in black and mean percentages listed in the adjacent tables. **(C)** Automated clustering of HLA-DR^+^ cells in the choriodecidua, merged tsne of saline (*n* = 4), LPS (*n* = 5), and blockade (*n* = 5). Clusters were identified by relative intensity of each antibody in the heatmap. Clusters that changed with treatment are outlined in black and mean percentages listed in the adjacent tables. *P < 0.05 on Kruskal–Wallis testing.

We next clustered on T cells specifically within the choriodecidua (Figure 4A) and found 18 unique populations. CD4^+^ clusters were separated based on CXCR3 expression and Treg phenotypes (CD25^+^CD127^lo^) (Figure 4A). Based on the expression of CCR7 and CD45RA (Figure 4A) we observed naïve (cluster 2), central memory (CM) (clusters 1, 16, 18), and effector memory (EM) (clusters 2, 4, 7, 9, 15) clusters to be present in the choriodecidua. In contrast, only EM phenotype was detected within the 7 clusters of CD8 cells identified. We report no alterations in T-cell frequencies between the three groups except for a trend (*P* = 0.08) toward decreased cluster 13 (EM CD8) in LPS-treated animals that was TNFα dependent (Figure 4A).

### LPS-Induced IA Inflammation Alters CD56^hi^ NK Cells

Decidual natural killer cells (dNK) are a specialized subset of NK cells that are phenotypically distinct from peripheral blood NK cells and marked CD56^superbright^ (37, 38). dNK cells have been implicated as critical cells in healthy and diseased pregnancies that play diverse roles from implantation to parturition [summarized in (39)]. To assess the NK cell compartment in the choriodecidua, we clustered on NK cells (Figure 4B). Clusters were classified as CD56^lo^, CD56^mid^, or CD56^hi^ (likely canonical dNK). We found cluster 20 (CD56^hi^CD16^+^CXCR3^+^) was trending toward increase in blockade animals alone (Figure 4B). While cluster 11 (CD56^hi^CXCR3^+^) was significantly reduced in IA LPS-exposed monkeys, and partially corrected in blockade. This finding is consistent with reports in mice that show administration of LPS at 9.5 days post-coitum leads to a decrease in NK cells in the uterus (40).

### Choriodecidual HLA-DR^+^ Cells Increase With LPS Independently of TNFα

Next, we analyzed choriodecidual HLA-DR^+^ cells (Figure 4C), representing APCs including B cells and MDCs. HLA-DR^+^ is also a marker of activated T cells (41, 42) and thus these cells were detected in the HLA-DR^+^ compartment as well. IA LPS treatment significantly altered the HLA-DR^+^ non-T-cell landscape with two populations of monocytes (clusters 18 and 20), one population of DCs (cluster 10), and one population of B cells (cluster 7) that were elevated in a TNFα-independent fashion (Figure 4C). Furthermore, one population of monocytes (cluster 9) was increased with LPS in a TNFα-dependent manner. This contrasts with our findings from the CD45^+^ clustering in which cluster 2 monocytes (HLA-DR^+^CD4^+^CD45RA^+^CXCR3^−^) were depleted with LPS treatment (Figure 3E). We determined that this cluster 2 population of monocytes is two distinct clusters when only HLA-DR^+^ cells are analyzed (clusters 3 and 4) and confirm our observations with a trend toward a decrease of these monocytes in LPS-treated animals (Figure 4C). The two monocyte populations that expanded the most with LPS treatment were CXCR3^+^ while the monocyte populations that were reduced after LPS exposure were CXCR3^−^ (Figure 4C). CXCR3 on monocytes has been shown to be upregulated during infection and inflammation (43), suggesting that either the endogenous decidual monocytes already present upregulated their CXCR3 expression or there was an influx of new CXCR3^+^ monocytes on LPS treatment.

### Signaling in Choriodecidual Immune Cells Is Not Altered in Our Experimental Window

To evaluate what signaling pathways are altered with IA LPS in the choriodecidua, we used intracellular antibodies against phosphorylated (p) proteins as a surrogate of STAT/MAP kinase/TCR/mTOR/CREB/IRAK signaling families (Table S3). Overall, at baseline we observed high phosphorylation of CREB in all CD45^+^ cells (Figure S2A) with highest levels of pCREB in APCs (Figures S2B,C,F). Additionally, we observed moderate levels of phosphorylation in the MAP kinases (ERK and p38), mTOR (S6), and IRAK4 pathways (Figure S2A), all critical to cell regulation. Moreover, we detected low levels of phosphorylation of STATs 1, 3, and 6. However, in the time analyzed, we report no significant change in any phosphorylation levels between the three treatment groups within total CD45^+^ cells (Figure S2A) and individual subtypes (Figures S2B–F).

### LPS Induces Phosphorylation of STAT1 and ZAP70 in a TNFα-Dependent Manner in the Villi

**Figure 5:**
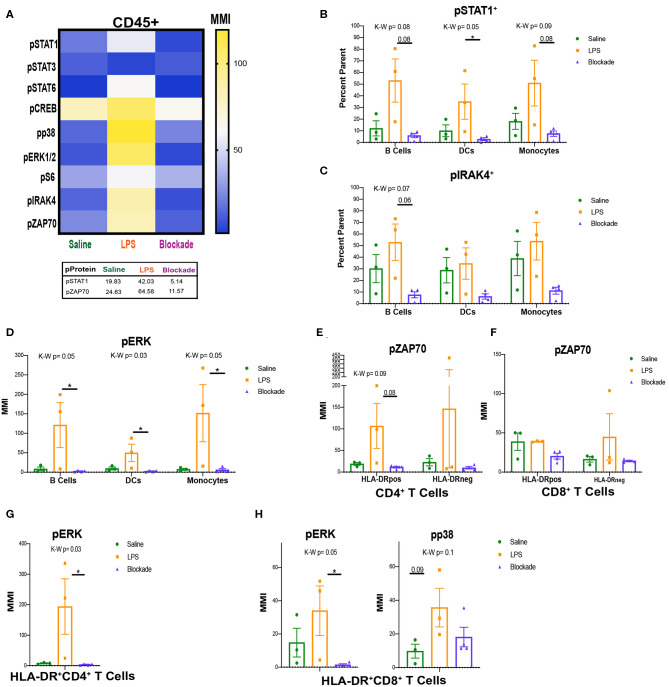
Signaling in villi and activated (HLA-DR^+^) T cells. **(A)** MMIs of phosphorylated proteins in all CD45^+^ cells from the villi as a heatmap. Average MMIs from the most significantly altered proteins between treatments are listed in the table below. **(B**–**E)** MMI or percent of parent of phosphorylated proteins in each APC subset. **(F**–**H)** MMI of phosphorylated proteins in HLA-DR^+^ T cell subsets. Kruskal–Wallis values are listed at the top of each graph. *Post hoc P*-values are listed as lines connecting respective comparisons. *P < 0.05.

When we analyzed protein phosphorylation of all CD45^+^ cells in the villi, we detected a trend toward upregulation of nearly all phosphorylated proteins on IA LPS treatment (Figure 5A). We also confirmed that villi T cells signaled through pS6, as expected (44) after PMA/Ionomycin stimulation (Figure S3A). In saline-treated animals, we observed high levels of pCREB and moderate levels of pS6 and pZAP70 with little baseline phosphorylation of other proteins (Figure 5A). When exposed to LPS, all proteins had a trend toward a TNFα-dependent increase in phosphorylation except for STAT3. The two proteins that were the most sensitive to prenatal exposures were pSTAT1 and pZAP70 (*P* = 0.09). On LPS treatment, there was a trending increase in both pSTAT1 MMI in CD45^+^ cells (Figure 5A) and a significant increase in the percent of cells with pSTAT1 (Figure S3B) that was TNFα dependent. Moreover, the steady-state pSTAT1 phosphorylation in the saline-treated animals was also TNFα dependent, as the blockade treatment reduced the overall pSTAT1 level below saline (Figure 5A). Additionally, we detected a trend toward an increase of pZAP70 in CD45^+^ cells in LPS-treated monkeys that was reduced in the blockade group. Like pSTAT1 levels, the blockade group also had reduced pZAP70 levels compared to the saline controls (Figure 5A). ZAP70 is phosphorylated on activation of the T-, NK-, and B-cell receptors (45, 46). The effect of LPS on ZAP70 is likely indirect given that LPS cannot directly activate the TCR. To further investigate if phosphorylated ZAP70 may be the result of TCR activation, we examined if APCs and activated (HLA-DR^+^) T cells specifically were responding to prenatal exposure of LPS.

### STAT1, IRAK4, and ERK MAP Kinase Are All Activated in LPS-Exposed APCs

To uncover which cells the pSTAT1 and pZAP70 signatures were originating from we began analyzing immune subtypes individually. When looking at APCs, we report that IA LPS exposure results in a higher frequency of cells that have a trending increase of pSTAT1 in all three of the APC subsets (MPCs, DCs, and B cells), DCs significantly so (Figure 5B). This is also seen by MMI in DCs (Figure S3C). This finding further supports that the STAT1 pathway is activated in the villi, and that TNFα signaling is required for its activation. In addition to STAT1, we also detected a trend toward increased phosphorylation of IRAK4 in the LPS compared to blockade groups in B cells (Figure 5C). IRAK4 becomes phosphorylated on activation of TLR4. LPS is sensed through TLR4 on the surface of immune cells, and thus the trend of increased pIRAK4 in LPS-treated animals may be due to direct recognition of LPS by villous B cells.

Further supporting activation of APCs with IA LPS, we detected alterations in MAP kinase signaling (Figure 5D). Specifically, within B cells, DCs, and monocytes we report significant increases in ERK1/2 phosphorylation by MMI that was TNFα dependent (Figure 5D). ERK1/2 is downstream of the IRAK4 signaling pathway, and their phosphorylation indicates that in addition to having inducible STAT1 signaling, villi APCs may be directly detecting LPS via TLR4/IRAK4 and then activating further downstream pathways.

### Activated T Cells Signal Through ZAP70 During IA Inflammation

After we found that APCs were the source of the pSTAT1 signature that we observed in Figure 5A, we next investigated what cells were phosphorylating ZAP70. As ZAP70 is phosphorylated downstream of TCR activation by APCs, we began looking at pZAP70 in activated T-cell populations. We report that in CD4 HLA-DR^+^ T cells, there was a trend toward reduction in pZAP70 in blockade vs. LPS groups, but no changes in the HLA-DR^−^ population (Figure 5E). These results are consistent with our observations of higher pZAP70 in LPS and lower pZAP70 in blockade in the total CD45^+^ population (Figure 5A). CD8 T cells, in contrast, did not have any alterations in pZAP70 between treatments, but higher pZAP70 MMI was detected in saline animals in HLA-DR^+^ vs. HLA-DR^−^ subsets (Figure 5F). Additionally, we observed significant increases in the map kinases: pERK in CD4 (Figure 5G) and pERK and pp38 (trend *P* = 0.09) in CD8 (Figure 5H), which are downstream targets in multiple pathways including TCR activation. These findings suggest that T cells in the villi respond to antigens through the TCR, and that this response is inhibited when TNFα signaling is inhibited.

### Immune Cells From the Placental Villi Alter Cytokine Production With IA LPS

**Figure 6:**
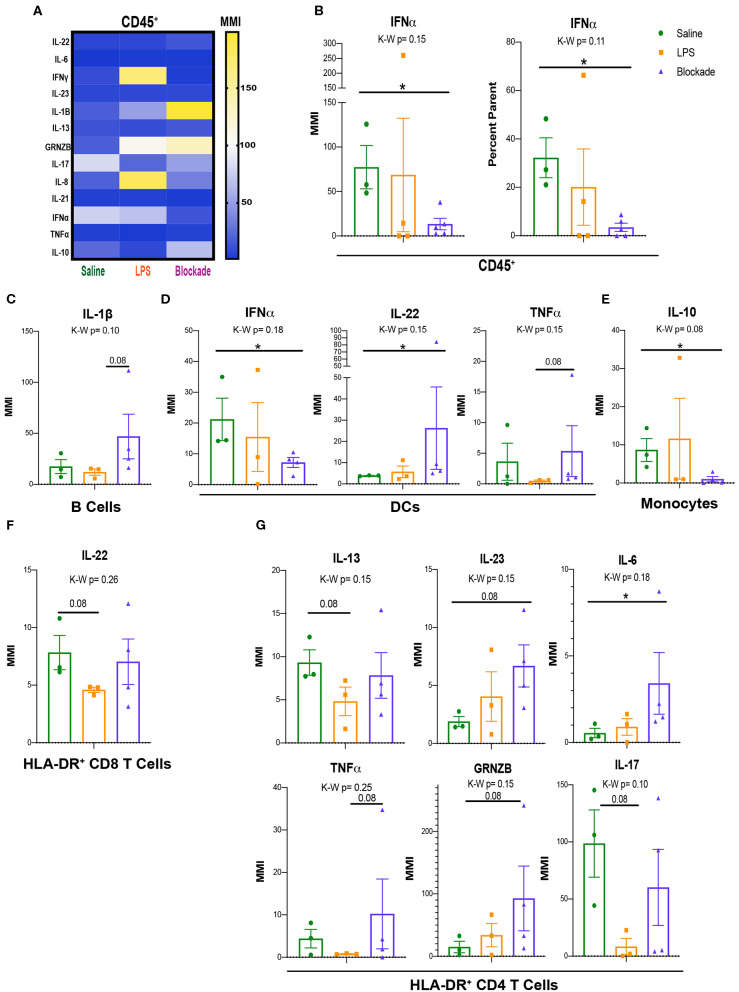
Signaling and cytokine production in villi APCs. **(A)** MMIs of cytokines in all CD45^+^ cells from the villi as a heatmap. The most significantly altered cytokines between treatments are listed in table below. **(B)** IFNα MMI comparisons between treatment groups. **(C**–**E)** MMI of cytokines in each APC subset. **(F,G)** MMI of cytokines in HLA-DR^+^ T cell subsets. Kruskal–Wallis values are listed at the top of each graph. *Post hoc P*-values are listed as lines connecting respective comparisons. *P < 0.05.

Once we determined that leukocytes in the placental villi had differential signaling at baseline during our experimental window, we sought to determine if their cytokine profiles differed with IA LPS. As such, we used a cytokine CyTOF panel (Table S3) to evaluate differences in cytokine production between saline- and IA LPS-treated monkeys. We report that CD45^+^ cells from the villous placenta produce high amounts of IL-17A and IFNα under homeostatic conditions (saline-treated animals) (Figure 6A). After *in utero* exposures we observed alterations in production of multiple cytokines, particularly granzymeB (GRNZB), IFNγ, IL-8, and IL-17, that were not statistically significant (Figure 6A). However, we observed a significant decrease in both MMI and percent positive cells for IFNα in the blockade group when compared to the saline-treated group (Figure 6B). To determine which immune cell subsets these cytokine signatures were originating from we examined major subsets of immune cells individually (Figure S4).

### IA Inflammation Induces Alteration of Cytokine Production by Villi APCs

Similar to our approach to uncovering signaling pathways within APC subtypes (Figure 5), we assessed cytokine production by APCs. Although we saw no differences when all three treatment groups were compared together, represented by the Kruskal–Wallis value, we found multiple cytokines altered within the blockade group when each treatment was compared individually (Figures 6C–E). Consistently, IFNα and IL-17A were the highest expressed cytokines in all three of the major APC subsets, B cells, DCs, and monocytes in saline-treated monkeys (Figures S4A–C). In B cells, we report a trend toward an increase in IL-1β in blockade compared to LPS (Figure 6C), indicating that with TNFα blocked *in vivo*, B cells produced higher amounts of IL-1β. DCs have a significant increase in IL-22 and a significant decrease in IFNα in an LPS-independent, but TNFα-dependent manner (Figure 6D). Additionally, DCs had a trending increase in TNFα production in blockade-treated animals compared to LPS-treated animals (Figure 6D). Blockade treatment also significantly reduced IL-10 production in monocytes (Figure 6E).

### Activated T-Cell Cytokine Production Is Sensitive to Prenatal IA LPS Exposure

As previously noted, we observed evidence of activation of T cells via increased HLA-DR expression (Figure 2F) and phosphorylation of the TCR pathway (Figures 5E–H). We examined cytokine production in the HLA-DR^+^ T cells to determine the cytokines produced by activated T cells. In both CD4 and CD8 T cell subsets, we observed lower cytokine production in HLA-DR^−^ T cells in saline-treated animals than HLA-DR+ counterparts (Figure S4D), further confirming that these HLA-DR^+^ T cells are activated. In saline-treated animals, HLA-DR^+^ T cells produce high amounts of IFNα and IL-17A by MMI (Figure S4D). In CD8 T cells, we report that only IL-22 was trending toward reduced levels (*P* = 0.08) in IA LPS-treated animals compared to saline-treated controls that was reversed with TNFα blockade (Figure 6F). In contrast, cytokine production by CD4 cells was more affected, where six cytokines—IL-13, IL-23, IL-6, TNFα, GNZB, and IL17A—had trending altered by LPS/blockade treatment (Figure 6G). These findings are congruent with recent work showing alterations of multiple cytokines in amniotic fluid of pregnant mice injected with anti-CD3 antibody (47). Overall, we have shown that activated (HLA-DR^+^) T cells have multiple phosphorylated components of the TCR pathway and change their cytokine profile in inflamed *in vivo* environments.

### Villi Tregs Are Depleted and Have an Altered Cytokine Profile

**Figure 7:**
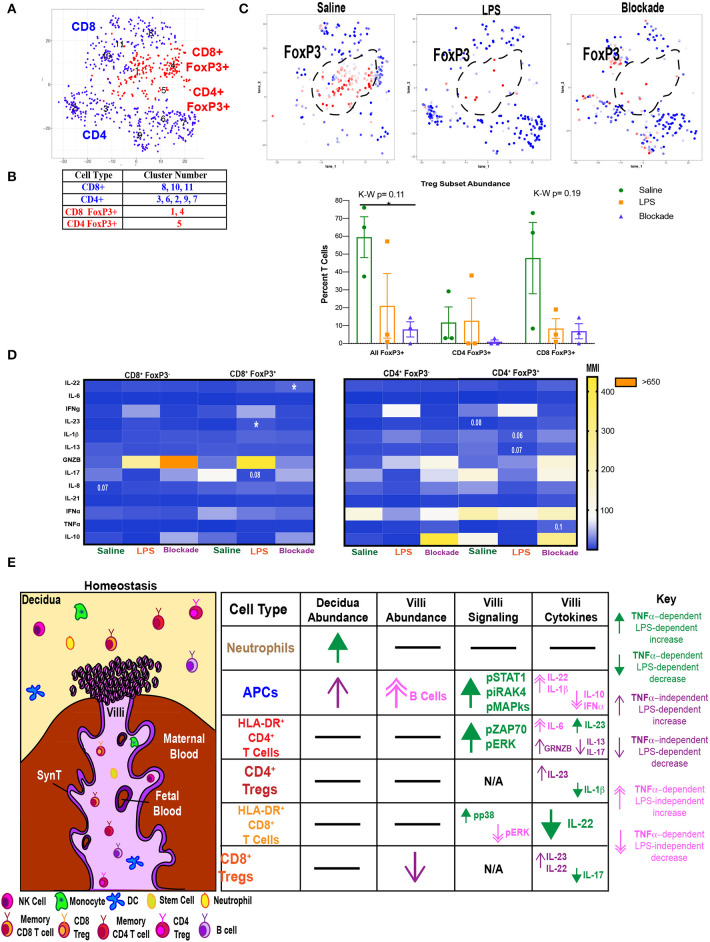
Abnormal T regulatory cell responses under intra-amniotic inflammation are exaggerated with TNFα inhibition. **(A)** Automated clustering of on T cells from cytokine panel (*n* = 3 per group). **(B)** Populations were identified based on antibody intensity. **(C)** FoxP3 expression CD4 and CD8 T cells, FoxP3+ cells are outlined in black and quantified as a percent of all T cells. Kruskal–Wallis values are listed at the top of each graph. *Post hoc P*-values are listed as lines connecting respective comparisons. *P < 0.05. **(D)** Heatmap of MMIs of cytokines in FoxP3^+^ and FoxP3^−^ T cells. Comparisons are made between treatment groups within each subset of T cells. Significance was determined using Kruskal–Wallis comparisons. Comparisons listed on heatmaps are those with an overall Kruskal–Wallis *P* < 0.1 and are placed on the box of the group with the most different value from other treatments. **(E)** Summary of findings in this article. SynT, syncytiotrophoblast.

We next investigated if Tregs were affected by IA LPS treatment. To do this we clustered T cells using our cytokine panel based on surface marker and FoxP3 expression (Figure 7A) and identified clusters that were CD25^+^FoxP3^+^. We found that both CD4 (cluster 5) and CD8 (clusters 1 and 4) populations contained FoxP3^+^ cells (Figures 7A,B). There was a trend toward a reduction in all FoxP3^+^ cells in both LPS-treated and blockade groups (Figure 7C). When stratifying between T-cell subtypes it appears that the abundance of CD8 Tregs is more sensitive to treatment than CD4 Tregs (Figure 7C). We next examined cytokine production in Tregs (FoxP3^+^ CD4 or CD8 T cells) compared to effector T cells (FoxP3^−^ CD4 or CD8 T cells) (Figure 7D). In confirmation of our phenotypic cluster identification (Figure 7B), we observed higher IL-10 MMI in saline-treated animals in both CD4 and CD8 Tregs compared to effectors (Figure 7D). In CD8 Tregs, we saw LPS-independent, TNFα-dependent production of IL-22 (Figure 7D). CD8 Tregs also had a trend toward suppressed IL-17 in a TNFα-dependent manner when given IA LPS (Figure 7D). Moreover, we saw a significant rise in IL-23 production in IA LPS that was corrected and restored in blockade (Figure 7D). CD4 Tregs were also sensitive to LPS/TNFα inhibition, as we saw varying alteration trends of Il-1β, IL-13, GRNZB, and TNFα (Figure 7D). In summation, we showed a loss of CD8^+^ FoxP3^+^ cells in both LPS- and blockade-treated animals and a more proinflammatory cytokine profile in both CD4^+^ and CD8^+^ Tregs that were previously exposed to IA inflammation that is increased with TNFα inhibition.

In summary, we have shown that the choriodecidua and placental villi contain diverse and distinct immunological profiles under homeostatic conditions. Within each tissue there are changes in individual immune cell populations with IA LPS that is not universally corrected with blockade (Figure 7E). Moreover, villous immune cells are signaling in a TNFα-dependent manner when exposed to IA LPS. Specifically, we have revealed that villous APCs sense LPS through IRAK4 and elevate pERK, pp38, and pCREB, which are phosphorylated with TNFα signaling (Figure 7E). Activated APCs subsequently may activate T cells through the TCR pathway and ZAP70. These activated T cells then alter cytokine production, and this mis-regulation of cytokines may be the resultant of a reduction and abnormal cytokine production by Tregs.

## Discussion

There is a need for a better understanding into the mechanisms driving IA inflammation, as these signals have residual long-lasting maternal (48) and fetal (5, 6) effects. Controlling inflammation at the fetal–maternal interface, which is made up of three layers—the maternal decidua fetal membranes, and placental villi—is critical to preventing multiple pathologies in neonates and mothers not limited to placental dysfunction, preeclampsia, and spontaneous preterm labor. However, to date, most of the work involving inflammatory processes at the fetal–maternal interface have focused on the decidua and membranes with sparse data available on the role of placental villi in this process. We aimed to investigate what the immune landscape is in both the choriodecidua and the fetal villi, and whether these cells contribute to inflammation in a rhesus macaque model of LPS-induced IA inflammation. Additionally, we aimed to understand if LPS-induced inflammation is TNFα dependent and can be alleviated with TNFα blockade, as a potential therapeutic.

Using mass cytometry, we uncovered a complex immune landscape within the choriodecidua and placental villi. Moreover, we found that these two landscapes were distinct from one another. Both the choriodecidua and the villi house phenotypically diverse innate and adaptive immune cells. Furthermore, using panels targeting phosphorylated proteins and cytokines, we show that cells within the villous placenta respond to LPS in the LPS/TLR4 and STAT1 pathways during our experimental window and produce a wide variety of cytokines without *ex vivo* stimulation. Furthermore, we show that villous immune cells can be further stimulated to secrete cytokines on PMA/ionomycin stimulation. We also conclude that some but not all of these effects are TNFα dependent. When looking at the choriodecidua, we were unable to detect the same changes in phosphosignaling we observed in the villi; however, this is most likely reflective of a suboptimal time window.

Consistent with prior studies, we detected a diverse population of immune cells in the choriodecidua [reviewed in (49)]. Moreover, as previously described (8), we saw an elevation of presumptive neutrophils on IA LPS treatment that was corrected with TNFα blockade. Furthermore, IA LPS induced an increase in CXCR3^+^ monocytes in the HLA-DR^+^ compartment, consistent with prior work showing that CXCR3 upregulation on macrophages is beneficial in the context of inflammation (43). Collectively, our choriodecidual results suggest that inflammation within the choriodecidua on IA LPS injection is a tightly regulated response that involves individual immune cell population changes rather than global immune shifts.

Looking at nine signaling proteins, we found no differences in phosphorylation between any of our treatment groups in the choriodecidua. This contrasts with previous work showing that immune cells in the choriodecidua are highly active in this model (7, 8). In our model, LPS is administered and then tissues are harvested 16 h later. Alterations in cytokine production in the decidua were previously observed at this time point, but these are likely downstream of alterations in the signaling cascades we were specifically focusing on in this article. As such the lack of changes in the phosphorylation status in choriodecidual immune cells may secondary to (1) alterations in signaling cascades occurs prior to the time point of tissue collection in our experiments; (2) high baseline level of protein phosphorylation, making it challenging to detect any differences; or (3) other signaling pathways mediate inflammation in our model. Future experiments are needed to address these possibilities.

When turning to the villous placenta, we found a diverse immune landscape that was distinct from the choriodecidua. We report a robust population of monocytes within the villi reflective of the previously documented Hofbauer cell population. Hofbauer cells are placenta-resident macrophages found in many mammalian species and are of fetal origin (32). Furthermore, our results significantly build upon existing data by reporting diversity of immune cell populations including: T cells (14, 50), monocytes (32, 51), DCs, NK cells, neutrophils, and B cells within the villi of the third-trimester primate placenta. The detection of B cells in the villi is of particular interest. B-cell populations within the decidua are rare, reportedly encompassing only 1–2% of CD45^+^ cells at term, and can acquire pathologic phenotypes in cases of preterm labor (36). The greater abundance B cells in the villi within the control saline-treated group suggests that B cells may play a villi-specific function in maintaining pregnancy and should be further investigated. Furthermore, we identified a group of CD45^+^clusters that were major lineage^−^ CD10^+^ and not present in the choriodecidua. CD10 has been shown to be expressed on hematopoietic stem cells (52). Additionally, the placenta is a site of hematopoiesis *in utero* (34), and based on these observations we propose that this niche of cells represents hematopoietic stem cells. Similar to the trends we observed in the choriodecidua, LPS treatment did not alter the relative composition of major immune subtypes in the placental villi, and instead caused alterations of specific immune populations. As such, studies should focus on investigating the role of specific immune populations such as CD45RA^+^ CXCR3^−^ monocytes, rather than investigating all monocytes together.

Immune cells in the villi upregulated phosphorylation of multiple proteins including STAT1 in a TNFα-dependent manner. Our data show that the source of pSTAT1 upregulation could be villi immune cell–derived IFNα. However, there are other sources of IFNs in the placenta (53, 54) that we did not directly address. When we studied APCs specifically, we observed the same trends in pSTAT1 that we observed at a global CD45^+^ level, showing that this was an APC-associated signature. It would be interesting to study the impacts of trophoblast vs. APC-secreted interferons on pSTAT1 signaling on all villi CD45^+^ cells.

Additionally, we saw alterations in pIRAK4 levels, a critical component in the sensing of LPS. The lack of significance we reported in saline vs. LPS groups is most likely attributed to our small number of animals in each group. However, we did detect a significant reduction in pIRAK4 in the blockade-treated animals. This finding supports the hypothesis that TNFα is required for placental villi APCs to sense LPS. Interestingly, it is reported that the amniotic fluid contains inhibitors to LPS-mediated TLR4 signaling in the fetal mouse intestine (55). It is plausible that addition of TNFα-blocking antibodies into the amniotic fluid is acting in a similar manner as these suppressive factors.

In our villi T-cell analysis, we report only T cells with central or effector memory surface markers in the villi. These findings are consistent with four recent studies showing phenotypical memory T cells in the second-trimester fetal intestine (15–17, 20) and activated T cells within the placental villi of preterm deliveries (22). Although several recent studies have shown that memory T cells can be found in the cord blood (19) and fetal intestine (15–17, 20) presence of memory T cells in the primate placental villi has not been previously described. Additionally, we saw upregulation of HLA-DR and increased pZAP70 among specific T-cell clusters in T-cell subsets indicating activation of T cells through the TCR. One interesting finding was that HLA-DR^+^CD4^+^ T cells in the blockade-treated group produced cytokines not commonly made by T-helper cells such as granzyme B. However, granzyme B^+^ CD4 T cells have also been reported in the fetal small intestine (16) and in the decidua of mothers with preterm labor (47). These findings suggest that TNFα signaling in the villi is required to keep T cells from secreting proinflammatory cytokines in healthy pregnancies.

Similar to effectors, we saw abnormal expression of cytokines in Tregs in both of our treatment groups. Our findings are like Rueda and colleagues' report of proinflammatory FoxP3^+^ CD4 T cells in fetal blood, spleen, thymus, and lung using a similar model of rhesus IA inflammation. These inflammatory Tregs also could not be corrected with prior administration of anti-IL-1 antibody, congruent with our findings using TNF blocking antibody (26). Our findings of FoxP3^+^ CD8 T cells add to the growing body of literature supporting the importance of CD8 Tregs as critical players under both homeostatic and proinflammatory environments (56). Furthermore, the presence of CD8 T cells with an anti-inflammatory cytokine profile has been shown to be present in the decidua and contribute to maintain healthy pregnancies (57). It is possible the Tregs detected by our study and by Rueda and colleagues were generated at the same time in the fetus and then have trafficked to the blood, spleen, thymus, lung, and placental villi. Our findings add to this field and confirm that these abnormal Tregs are at the fetal–maternal interface during IA inflammation and potentially orchestrate an inflammatory response throughout the amniotic cavity.

As previously stated, we observed that some, but not all LPS-induced modulations in immune cell abundance and signaling were reversed with TNF blockade. These findings suggest that some phenotypes of IA inflammation within the villi such as phosphorylation of the TCR pathway proteins are dependent on TNF signaling, while other phenotypes such as depletion of the Treg population are mediated through alternative cytokine pathways. Moreover, it is known that other cytokines in addition to TNFα are elevated with IA inflammation (58), and future studies investigating the role of inhibition of these may uncover the mechanisms behind the TNFα-independent phenotypes reported in this study.

The main limitations of our study were the relatively small sample size of each treatment group. Including more animals per treatment group would be helpful in determining if many of the trends we report were significant, but this was not feasible in the current experiment. However, the use of primate models is imperative to furthering our understanding of the etiologies of preterm birth. Rodent models do not closely replicate the anatomy of the human fetal–maternal interface, nor the triggers of parturition (24). Another limitation is that our samples were exposed to unavoidable overnight transport between facilities. However, we did observe that cells could be stimulated with PMA as expected, suggesting that signaling cascades could still be initiated post-transport. It would be interesting to know the anatomical locations within the placental villi of the immune populations studied that can be accomplished by techniques such as imaging mass cytometry (59). A larger cohort of animals as well as the addition of a group that received just the TNFα blockade without LPS exposure would be valuable to answer further questions about the effects of TNFα signaling on the immune landscape of both the choriodecidua and placental villi; unfortunately these tests were not feasible for this experiment.

In conclusion, we report there is a complex and distinct immune landscape at the fetal maternal interface during homeostasis and IA inflammation in both the choriodecidua and villi of rhesus macaques. Our data show that the immune profile of these two tissues is distinct from one another and the villi should be considered in future immunological studies at the fetal–maternal interface. This study has broad implications for increasing our knowledge of primate placental biology and immunological drivers of IA inflammation.

## Data Availability Statement

The datasets generated for this study are available on request to the corresponding author.

## Ethics Statement

The animal study was reviewed and approved by IACUC at University of California, Davis.

## Author Contributions

SK, CC, and LK conceived and funded the experiments. JT, SS, and CM carried out all villi-specific experiments. PP and MC stained all choriodecidual samples and performed placental preparation. JT performed analyses for both villi and choriodecidua experiments supervised by LK. All authors contributed to the writing and editing of the manuscript.

## Conflict of Interest

The authors declare that the research was conducted in the absence of any commercial or financial relationships that could be construed as a potential conflict of interest.
