# Supplementary material for: Immune Cells in the Placental Villi Contribute to Intra-amniotic Inflammation
Source: Front Immunol. 2020 May 22;11:866. doi: 10.3389/fimmu.2020.00866 (PMC7256198; doi:10.3389/fimmu.2020.00866)
Supplement: Supplementary file 1 [file Data_Sheet_1.PDF]

Table S1. Description of animals included in study

Animal ID	Maternal Age (years)	Maternal Weight (Kg)	Gestational Age at Birth (days)	Fetal Weight (Kg)	Fetal Gender
**Saline IA**					
423	10	8.14	132	.351	M
427	5	6.42	131	.223	M
**Saline IM**					
430	14	9.95	130	.282	M
432	15	9.96	129	.299	M
**LPS**					
429	16	11.18	132	.374	F
436	8	10.56	136	.420	F
437	13	11.31	136	.308	F
440	8	11.35	129	.281	M
442	8	6.78	130	.244	M
**Blockade**					
431	10	10.43	130	.389	F
438	11	12.58	134	.452	M
439	10	10.89	131	.339	F
441	14	10.7	131	.322	M
443	10	10.44	127	.183	F

Table S2. Samples omitted from individual experiments.

Sample	Figure	Omittance Justification
**Decidua**		
431	S2	Insufficient intracellular staining
432	S2	Insufficient intracellular staining
436	S2	Insufficient intracellular staining
437	S2	Insufficient intracellular staining
**Villi - Phospho**		
427	1, 2, 5, S3	Insufficient parent cell number
440	1, 2, 5, S3	Insufficient parent cell number
437	5, S3A,B	Insufficient intracellular staining
439	5, S3A,B	Insufficient intracellular staining
**Villi - Cytokine**		
427	6, 7, S4	Insufficient parent cell number
440	6, 7, S4	Insufficient parent cell number
436	6C-E, S4A-C	Insufficient parent cell number
437	6F-G; 7D, S4D	Insufficient parent cell number (7D FoxP3^{-} T cells)
438	6G, 7,S4D	Insufficient parent cell number (S4D HLA-DR+ CD4/CD8, HLA-DR- CD8)
439	6,7	Insufficient intracellular staining
442	7	Insufficient parent cell number (FoxP3+ T cells)

Table S3. Mass cytometry panels used. Antibodies in black were used in all panels. Antibodies in green were used in phospho-panel only. Antibodies in blue were used in cytokine panel only.

Metal Tag	Marker	Clone	Vendor
113In	CD88	P12/1	Bio Rad
115In	CD11c	IM7	Bio Rad
141Pr	CD86	37301	Novus Biologicals
142Nd	CD19	HIB19	BioLegend
143Nd	HLA-DR	L243	Fluidigm
145Nd	CD16	3G8	BioLegend
146Nd	CD8a	RPA.T8	BioLegend
147Sm	CD10	HI10a	BioLegend
148Nd	CD45	D058-1283	BD Biosciences
149Sm	CD25	2A3	Fluidigm
150Nd	IL-22	22URTI	Fluidigm
151Eu	CD56	NCAM16.2	BD Biosciences
152Sm	CD14	M5E2	BioLegend
153Eu	CD45RA	HI100	BioLegend
154Sm	CD38	HIT2	BioLegend
155Gd	CD27	L128	Fluidigm
156Gd	IL-6	MQ2-13A5	BioLegend
158Gd	CD20	2H7	BD Biosciences
159Tb	CCR7	G043H7	Fluidigm
160Gd	pSTAT1<br>IFNγ	58D6<br>4S.B3	Cell Signaling Technologies<br>Biolegend
161Dy	IL-23p19	23dcdp	Fluidigm
162Dy	IL-1B	H1b-27	BioLegend
163Dy	CD183	G043H7	Fluidigm
164Dy	pZAP70<br>IL-13	Y319<br>JES10-5A2	Cell Signaling Technologies<br>BioLegend
165Ho	pCREB<br>FoxP3	87G3<br>PCH101	Fluidigm<br>eBiosciences
166Er	pp38	D3F9	Cell Signaling Technologies
167Er	p44/42MAPK (ERK1/2)<br>GranzymeB	D13.14.4E<br>GB11	Cell Signaling Technologies<br>BioLegend
168Er	CD127	A019D5	Fluidigm
169Tm	pSTAT3<br>IL-17a	D3A7 XP R<br>BL168	Cell Signaling Technologies<br>BioLegend
170Er	IL-8	6217	R&D Systems
171Yb	CD68	Y1/82A	Fluidigm
172Yb	IL-21	3A3-N2	BioLegend
173Yb	pIRAK4<br>IFNα	D6D7<br>MAR1-583	Cell Signaling Technologies<br>BioLegend
174Yb	CD4	OKT4	eBiosciences
175Lu	pS6<br>TNFα	D57.2.2E XP R<br>Mab11	Cell Signaling Technologies<br>BioLegend
176Yb	pSTAT6<br>IL-10	D8S9Y<br>JES3-19F1	Cell Signaling Technologies<br>BioLegend
209Bi	CD3	Sp34.2	BD Biosciences

Supplemental Figures

Figure S1

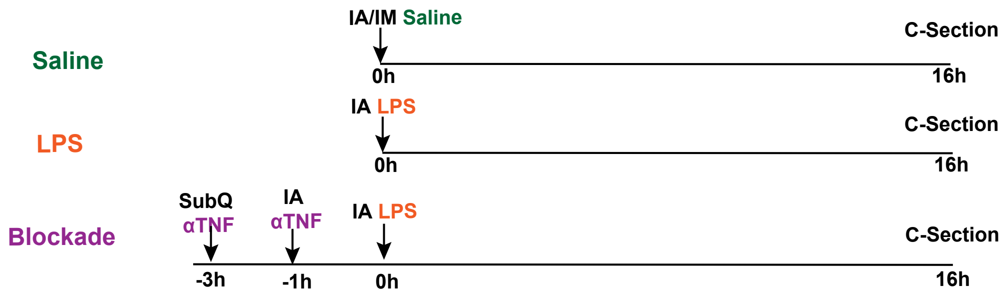

Figure S1. Rhesus model of intra-amniotic inflammation. Pregnant rhesus monkeys at 80% gestation were divided into three groups. Saline grouped animals received either intra-amniotic (IA) (n=2) or intra-muscular (IM) (n=2) and then were delivered via Cesearean-section (C-section) 16 hours later. LPS (n=5) grouped animals received IA LPS and were delivered via C-section 16 hours later. Blockade (n=5) grouped animals received subcutaneous (SubQ) anti-TNFα (αTNF) antibody at -3 hours then IA αTNF at -1 hours prior to IA LPS at 0 hours and were delivered via C-section 16 hours later.

Figure S2

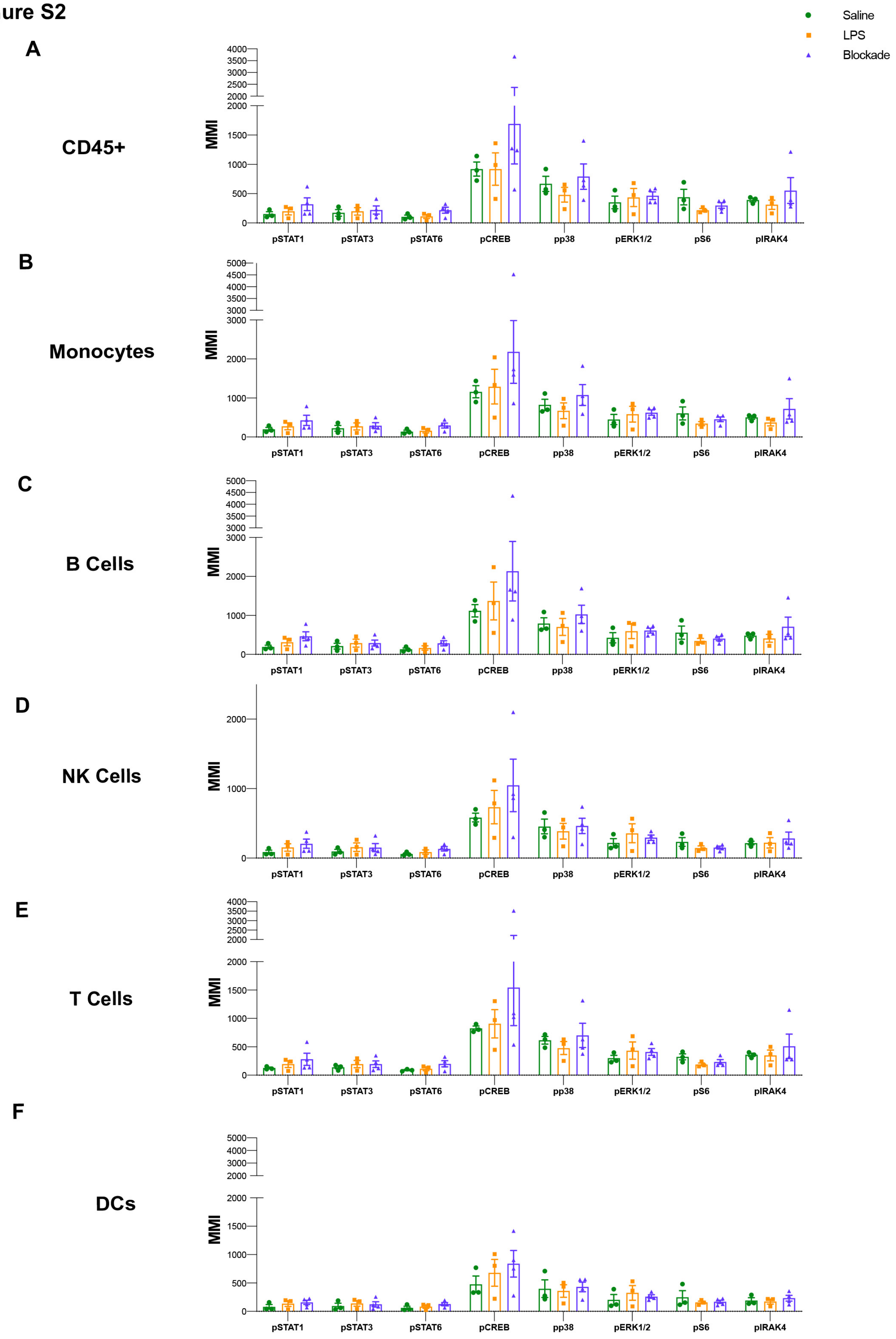
Saline
LPS
Blockade
A
CD45+
B
Monocytes
C
B Cells
D
NK Cells
E
T Cells
F
DCs
MMI
pSTAT1
pSTAT3
pSTAT6
pCREB
pp38
pERK1/2
pS6
pIRAK4

Figure S2. Signaling in choriodecidual immune subsets. Antibodies against phosphorylated proteins were used to evaluate signaling pathways in choriodecidual immune cells. **(A-F)** Total immune cells **(A)** and specific immune subsets **(B-F)** were 2D gated and Mean Metal Intensities (MMIs) were calculated and compared among treatment groups. Saline (n=3), LPS (n=3) blockade (n=4) after samples with insufficient staining or parent cell numbers were omitted (TableS2). No significant differences were detected among any groups when all three groups were compared (Kruskal-wallis values) or when individual treatments were compared among each other using post-hoc testing. P value > 0.05 was used as significant.

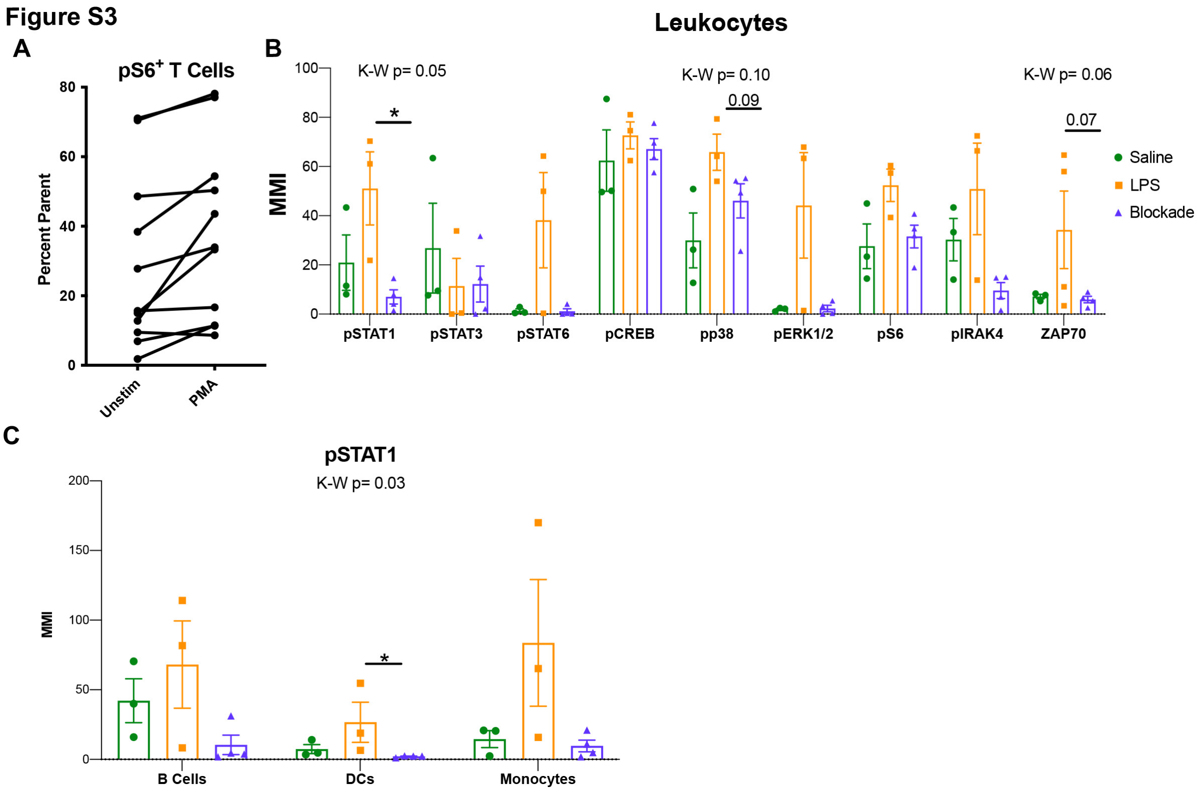

Figure S3 Phospho-signaling in placental-villi derived immune cells. Antibodies against phosphorylated proteins were used to evaluate signaling pathways in villi immune cells. **(A)** Validation that villi immune cells were still functional post transport. Percent of all T cells expressing pS6 in unstimulated and PMA/ionomycin stimulated conditions. Lines connect matched unstimulated-PMA stimulated samples from the same monkey. Saline (n=3), LPS (n=3) blockade (n=4) all combined on same graph **(B-C)** Total immune cells **(B)** and antigen-presenting cells (APCs) **(C)** were 2D gated and Mean Metal Intensities (MMIs) were calculated and compared among treatment groups. Saline (n=3), LPS (n=3) blockade (n=4) after samples with insufficient staining or parent cell numbers were omitted (TableS2). Kruskal-Wallis comparisons of all three groups are listed at the top of each group of comparisons, individual post hoc comparisons between each individual group are marked with lines. * = p-value <0.05.

Figure S4

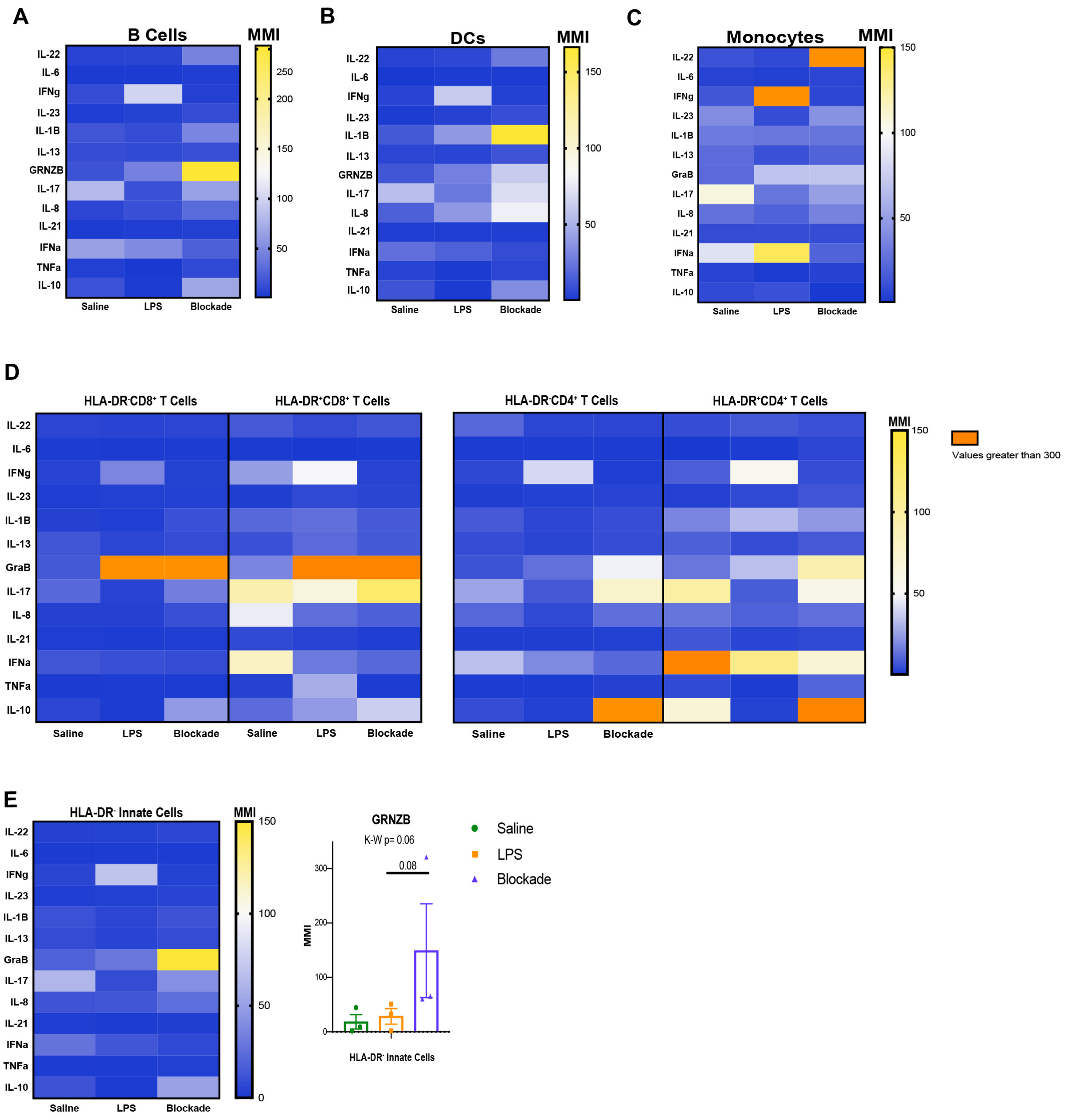

Figure S4 Cytokine production in villous immune subsets. Antibodies against cytokines were assessed in villi immune cells. **(A-C)** antigen-presenting cells comprised of **(A)** B cells **(B)** DCs and **(C)** monocytes were 2D gated and Mean Metal Intensities (MMIs) were calculated and displayed in heatmap form saline (n=3), LPS (n=3) blockade (n=4). Cytokines that were significantly altered in each group are displayed in Figure 6. **(D)** CD8 HLA-DR^{+} saline (n=3), LPS (n=3) blockade (n=3) and HLA-DR^{-} CD8 T cells saline (n=3), LPS (n=3) blockade (n=3) and CD4 HLA-DR^{+} T cells saline (n=3), LPS (n=3) blockade (n=3) and HLA-DR^{-} CD8 T cells saline

(n=3), LPS (n=3) blockade (n=4). Cytokines that were significantly altered in each group are displayed in Figure 6. **(E)** HLA-DR^- innate cells saline (n=3), LPS (n=3) blockade (n=3). Statistically trending cytokine granzyme B illustrated graphically.
